# Hybridization, polyploidization, and morphological convergence make dozens of taxa into one chaotic genetic pool: a phylogenomic case of the *Ficus erecta* species complex (Moraceae)

**DOI:** 10.3389/fpls.2024.1354812

**Published:** 2024-03-26

**Authors:** Xiaomei Wang, Shuai Liao, Zhen Zhang, Jianhang Zhang, Li Mei, Hongqing Li

**Affiliations:** ^1^ School of Life Sciences, East China Normal University, Shanghai, China; ^2^ Key Laboratory of Plant Resources Conservation and Sustainable Utilization, South China Botanical Garden, Chinese Academy of Sciences, Guangzhou, China; ^3^ South China National Botanical Garden, Guangzhou, China; ^4^ College of Architecture and Urban Planning, Tongji University, Shanghai, China; ^5^ School of Life and Environmental Sciences, Shaoxing University, Shaoxing, China

**Keywords:** *Ficus erecta* complex, RADseq, plastomes, phylogeny, species delimitation

## Abstract

The *Ficus erecta* complex, characterized by its morphological diversity and frequent interspecific overlap, shares pollinating fig wasps among several species. This attribute, coupled with its intricate phylogenetic relationships, establishes it as an exemplary model for studying speciation and evolutionary patterns. Extensive researches involving RADseq (Restriction-site associated DNA sequencing), complete chloroplast genome data, and flow cytometry methods were conducted, focusing on phylogenomic analysis, genetic structure, and ploidy detection within the complex. Significantly, the findings exposed a pronounced nuclear-cytoplasmic conflict. This evidence, together with genetic structure analysis, confirmed that hybridization within the complex is a frequent occurrence. The ploidy detection revealed widespread polyploidy, with certain species exhibiting multiple ploidy levels, including 2×, 3×, and 4×. Of particular note, only five species (*F. abelii*, *F. erecta*, *F. formosana*, *F. tannoensis* and *F. vaccinioides*) in the complex were proved to be monophyletic. Species such as *F. gasparriniana*, *F. pandurata*, and *F. stenophylla* were found to encompass multiple phylogenetically distinct lineages. This discovery, along with morphological comparisons, suggests a significant underestimation of species diversity within the complex. This study also identified *F. tannoensis* as an allopolyploid species originating from *F. vaccinioide* and *F. erecta*. Considering the integration of morphological, molecular systematics, and cytological evidences, it is proposed that the scope of the *F. erecta* complex should be expanded to the entire subsect. *Frutescentiae*. This would redefine the complex as a continuously evolving group comprising at least 33 taxa, characterized by blurred species boundaries, frequent hybridization and polyploidization, and ambiguous genetic differentiation.

## 1 Introduction

Species complexes, as species groups characterized by overlapping morphologies and incomplete genetic differentiation, emerge through various speciation processes, including hybridization, polyploidization, introgression, and incomplete lineage sorting (Wang, 1998; Nosil et al., 2009; Stankowski and Ravinet, 2021). Given that speciation is a protracted and continuous phenomenon, the species within a complex often stem from diverse speciation events or stage of speciation processes. Therefore, linking microevolutionary mechanisms (such as polyploidization and hybridization) with macroevolutionary patterns (like speciation) is vital for a thorough understanding of their speciation and evolutionary histories (Nosil et al., 2009; Christie and Strauss, 2018; Pinheiro et al., 2018; Lu et al., 2022). However, phylogenetic and evolutionary interpretations based on morphology in species complexes frequently encounter uncertainties. This is due to intrinsic or extrinsic factors that may induce morphological variation resembling each other. Morphological traits are often continuous, but their diagnosis is limited by taxonomic thresholds. Additionally, the rapid, repeated evolution of adaptive phenotypes further complicates the situation (Fišer et al., 2018; Prata et al., 2018; Galtier, 2019). The paucity of reliable molecular markers at lower taxonomic levels further hinders the evolutionary inference within species complexes (Avise, 2012; Galtier, 2019). Moreover, challenges such as hybridization, incomplete lineage sorting, and polyploidization often result in that gene trees inadequately represent true species trees (Degnan and Rosenberg, 2006; Yang and Rannala, 2010; Villaverde et al., 2018). To address these issues, we intent to obtain whole-genome data, together with morphological, cytological, and ecological evidences. This multidisciplinary strategy may be pivotal in accurate species delimitation within a complex, elucidating the phylogenetic relationships of morphological variations or taxa.

Taxonomically complex groups (TCGs) often exhibit characteristics that complicate their classification into discrete species, such as uniparental reproduction, diverse ploidy levels, and hybridization. The genus *Ficus* (Moraceae) is a notable example of a TCG, primarily owing to its significant intraspecific variability (Ennos et al., 2005; Mahima et al., 2020). This genus is distinguished by extensive species diversity and a rich evolutionary history, especially influenced by specific symbiotic pollinators, leading multiple morphologically indistinct species complexes (Berg, 2007; Wang et al., 2016; Su et al., 2022). For example, the *F. obliqua*–*F. rubiginosa* complex consists of three species, all of which are pollinated by closely related fig wasps (Wiebes, 1994; Dixon et al., 2001). The *F. sarmentosa* complex, encompassing at least 21 climbing taxa adapted to limestone habitats, is characterized by widespread interspecific hybridization, resulting in highly convoluted phylogenetic relationships (Zhang et al., 2023). Similarly, the *F. auriculata* complex (Zhang et al., 2018) and the “hairy-fig” complex (Lu et al., 2016) both display extensive genetic sharing and morphological continua among their respective groups. The *F. petiolaris* complex, endemic to Mexico, is distinguished by its morphological continuity, leading to its classification as a single species, *F. petiolaris* (Piedra-Malagón et al., 2011). These instances underscore that classification within *Ficus* can be significantly influenced by frequent hybridization and convergent morphological trends.

The *F. erecta* complex, along with its allies, constitutes a complex species group within subsect. *Frutescentiae* of subg. *Ficus*, predominantly distributed across the subtropical regions of China. These taxa are characterized by their dioecious syconia, predominantly small shrub-like habit, the distinct Terminalia-branching pattern, and axillary figs (Berg et al., 2005). Their foliage is typically narrow and papery, and may exhibit variations in pilosity. The accurate taxonomic identification within this group encounters considerable hurdles due to the scarcity of comprehensive specimen sets (including diverse morphological forms, both genders, and syconia at different developmental stages) and pronounced morphological plasticity, which has notably hindered advancements in the taxonomic, systematic, and evolutionary ecological researches of this group. The most extensive study hitherto conducted by Lu et al. (Lu et al., 2017) employed nuclear genes (ITS+ETS) and simple sequence repeats (SSR) to analyze the phylogeny of approximately 30 taxa within this subsection. This seminal work delineated a core clade, with limited internal branch support and characterized by overlapping morphological variations, as the *F. erecta* complex, comprising 14 species and 3 cryptic species, culminating in 17 taxonomic entities (*F. abelii*, *F. boninsimae*, *F. erecta*, *F. fengkaiensis*, *F. formosana*, *F. fusuiensis*, *F. gasparriniana* var. *laceratifolia*, *F. gasparriniana* var. *viridescens*, *F. iidaiana*, *F. nishimurae*, *F. pandurata*, *F. periptera*, *F. pyriformis*, *F. sinociliata*, *F. stenophylla*, *F. tannoensis* and *F. vaccinioides*). However, extensive comparative morphological anatomical investigations have yet to discover distinctive unique features which are capable of reliably differentiating most species within the complex (Zhou and Gilbert, 2003; Gui, 2013; Gao, 2018). This issue is particularly evident in taxa exhibiting varietal morphological diversity, such as *F. gasparriniana* and *F. pandurata*. Besides, a series of studies have underscored the prevalence of hybridization and pollinator sharing events within the complex. Notably, early hybridization events have been identified between *F. erecta* and some species endemic to Ogasawara Islands in Japan, such as *F. boninsimae*, *F. nishimurae*, and *F. iidaiana*. Additionally, *F. erecta* is presumed to share pollinators with *F. tannoensis* and *F. vaccinioides* from Taiwan, as well as the more widely distributed *F. formosana* (Kusumi et al., 2012; Wachi et al., 2016; Su et al., 2022). Nevertheless, the phylogenetic relationships within the complex remain largely unresolved due to limitations in sampling adequacy or the paucity of informative genetic loci. This is particularly evident in the ambiguous relationships among the varieties of *F. gasparriniana*, the distinct genetic diversification of *F. stenophylla*, and the weak support for existing phylogenetic branches (Lu et al., 2017). Consequently, the delineation of species within the complex is still in need of a more robust and comprehensive approach.

In *Ficus*, typical diploid plants possess a chromosome count of 2n = 26, as established in prior studies (Ohri and Khoshoo, 1987; Guasmi et al., 2006). Within the *F. erecta* complex, the occurrence of polyploidy in multiple species has been documented, further complicating taxonomy and obscuring the understanding of interspecific phylogenetic relationships and evolutionary history. Notably, *F. iidaiana* has been identified as a tetraploid (Ono, 1990; Kusumi et al., 2012). Additionally, genome sequencing has indicated the existence of tetraploid individuals in the *F. erecta* population, with genome size approximating 590 Mbp (Shirasawa et al., 2020). The presence of such polyploidy events not only complicates taxonomy of the *F. erecta* complex but also obscures the understanding of the interspecific phylogenetic relationships and its evolutionary history.

Chloroplast genomes, functioning as super-barcodes, are pivotal for species identification and are instrumental in discovering new taxa and cryptic species (Ji et al., 2019; Liu et al., 2021). Additionally, reduced-representation genomic techniques, such as restriction-site associated DNA sequencing (RADseq), have been widely employed in phylogenetic studies, particularly for their efficacy in elucidating complex reticulate evolutionary events, including introgression and hybridization in closely related groups (Eaton and Ree, 2013; Eaton et al., 2015; Razkin et al., 2016; Maguilla et al., 2017; Paetzold et al., 2019). In this study, we employed skimming sequencing to acquire complete chloroplast genomes, complemented by single nucleotide polymorphisms (SNPs) derived from RADseq. This dual genomic approach is aimed at reconstructing a robust phylogenetic tree and then detect hybridization and introgression events for the *F. erecta* complex. Combining these phylogenomic analyses and extra chromosome ploidy detection, the study finally tried to figure out cryptic species, the extent of species differentiation, and interspecific phylogenetic relationships within the *F. erecta* complex. This comprehensive phylogenomic analysis of the *F. erecta* complex is pivotal not only for unraveling its complex evolutionary history but also for a broader understanding of plant speciation and evolutionary dynamics. Moreover, this study sets a precedent for the meticulous delineation of taxa within taxonomically challenging groups.

## 2 Materials and methods

### 2.1 Sampling strategy

**Figure 1:**
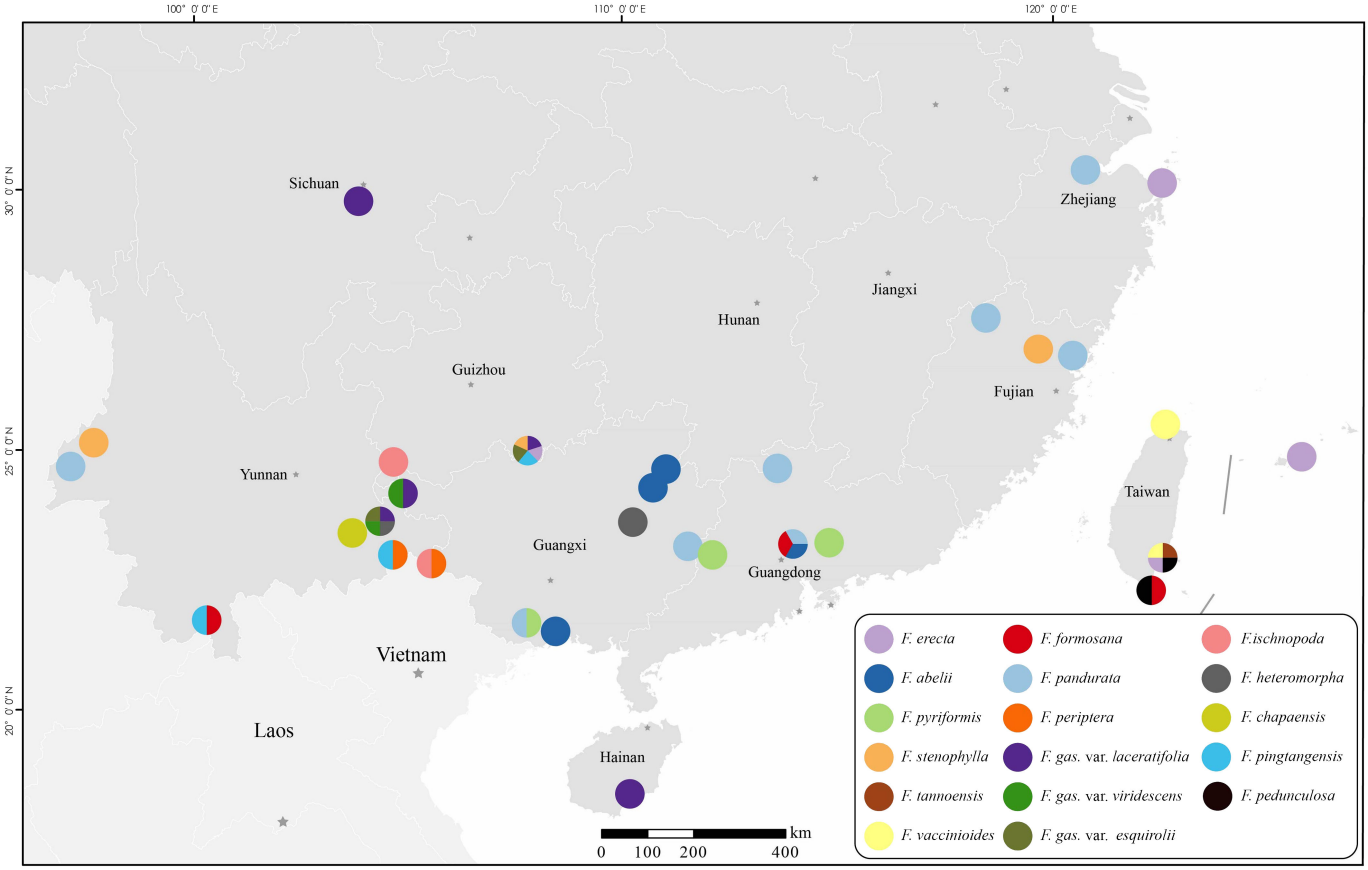
Geographical distribution map of the collected experimental materials.

The sampling strategy was meticulously devised to capture the extensive morphological diversity inherent in the complex. Particular attention was focused on species ambiguously classified as “the varieties of *Ficus gasparriniana*”, “*F. stenophylla*”, “*F. pandurata*” and specimens presenting morphological identification challenges within the complex. A representative set of 3–5 samples was collected for each species, with a minority represented by a single sample. Samples from species with restricted distributions were predominantly sourced from their respective type localities. This study successfully acquired samples from all taxa within the complex, with the exception of three cryptic species exclusively documented from type specimens in Southwest China and endemic species from Japan’s Ogasawara Islands. Informed by Lu et al.’s study (Lu et al., 2017), this study further incorporated representative samples from six related species, which are potential candidates for hybridization events within the complex. Evolutionary analyses were conducted on all samples, employing RADseq and genome skimming sequencing techniques. Individuals exhibiting markedly larger leaves and figs, indicative of potential polyploidy, were specifically selected for chromosome ploidy examination. The voucher specimens have been archived in the Herbarium of East China Normal University (HSNU). Detailed information on the samples is systematically cataloged in **Figure 1** and **Table S1**. (The sample name marked with a question mark, indicates that it is a cryptic species. Cryptic species refers to taxa that cannot be readily distinguished morphologically, yet evidence indicates they are on different evolutionary trajectories.).

### 2.2 DNA extraction, library construction, and sequencing

Total DNA was extracted utilizing a modified CTAB protocol (Doyle and Doyle, 1987), and its integrity was confirmed through agarose gel electrophoresis and detected by NanoDrop 2000 Spectrophotometer. Subsequently, DNA samples meeting quality standards were sent to BGI (Shenzhen, China) for library construction and sequencing. The chloroplast genome sequencing required a minimum DNA quantity of 1.5 μg with a concentration threshold of 20 ng/μL, while RADseq library sequencing stipulated a DNA minimum of 1 μg and a concentration of at least 12.5 ng/μL.

In the skimming sequencing approach, DNA purification was followed by fragmentation to generate short-insert (≤800 bp) paired-end reads. Sequencing tags facilitated the indexing of each sample’s genomic DNA for library construction. Whole-genome skimming sequencing was executed on the HiSeq™ 2000 platform (Illumina, San Diego, California, USA), yielding a minimum of 2 G of raw data per sample with 125 bp pair-end reads.

For RADseq library construction, EcoRI was selected based on pre-digestion experiments for enzymatic digestion, and sample-specific barcodes were employed to prepare RAD libraries. Sequencing was performed on the HiSeq™ 2000 platform (Illumina, San Diego, California, USA) utilizing PE150 paired-end sequencing, ensuring a data yield of no less than 1 G per sample. Sequencing data underwent quality control via FastQC (http://www.bioinformatics.babraham.ac.uk/projects/fastqc/) (Andrews, 2010). For subsequent assembly and analysis, read1 was chosen.

### 2.3 Data assembly

The initial trials with various assembly softwares revealed GetOrganelle as the superior tool in terms of assembly quality (Freudenthal et al., 2020). Thus, all samples were assembled using GetOrganelle (Jin et al., 2020), with *F.* religiosa (GenBank accession no. NC_033979) serving as the reference genome. The assembly parameters were meticulously optimized as followed: K-mer values were set to 55, 75, 95, 115, and 127 (Jin et al., 2020), with a word size of 85 and R at 15. For samples that did not automatically circularize, manual circularization was performed in Bandage v.0.8.0 (Wick et al., 2015), referencing other complete circular sequences from the same species to bridge gaps. Sequence alignments were executed using MAFFT v7.490 (Katoh and Standley, 2013) under default settings. Chloroplast genome annotation was conducted via Plastid Genome Annotator (PGA) software (https://github.com/quxiaojian/PGA) (Qu et al., 2019), using *F.* religiosa (NC_033979) (Bruun-Lund et al., 2017) and *F.* sarmentosa (NC_061976) (Zhang et al., 2022) as reference genomes. The tRNA genes’ boundaries were accurately determined using the tRNAscan-SE server (http://lowelab.ucsc.edu/tRNAscan-SE/) (Chan and Lowe, 2019). The whole plastid genome was aligned and used for phylogenetic analysis, with one of the two inverted regions and *psbZ*, and *psbZ*-*trnG*-*GCC* (The chloroplast genomes of three samples is incomplete, with one gap in this region.) removed.

Considering its robustness in processing indels and estimating heterozygosity (H) and sequencing error rates (E), ipyrad (Eaton and Overcast, 2020) was selected for RADseq data assembly using the single end of the paired-end sequences (R1) after control via FastQC. The data filtering was conducted in ipyrad through replacing bases with a Phred quality score (Q) below 33 with an ambiguous base (“N”), discarding reads exceeding 10% “N”. Clustering, based on sequence similarity, was conducted using UCLAST algorithm in USEARCH software tool (Edgar, 2010), followed by estimating individual hybrid rate (H) and sequencing error frequency (E) (Lynch, 2008), filtering out paralogous and highly repetitive sequences to attain consensus sequences. To mitigate the clustering values’ and sample coverage’s impacts on the phylogeny, we tested cluster_thread threshold of 0.85, 0.90, and 0.95, with 17 min_sample_loci gradients set from low to high under each threshold. This approach generated 51 sets of unlinked SNP datasets for the phylogenetic tree reconstruction. Consistency across phylogenetic tree topologies derived from all 51 datasets was observed, with only minor variations in shallow nodes and support values. The optimal data matrix for subsequent analyses was identified by calculating the Robinson-Foulds distance between different trees using RF.dist function in the phangorn package in R (Robinson and Foulds, 1981). The dataset with cluster_thread at 0.90 and min_sample_loci at 31 (i.e., c90min31), which exhibited the minimum Robinson-Foulds distance relative to others (**Table S2**), was selected for the phylogenetic tree construction and subsequent analyses (**Table S3**).

### 2.4 Phylogenetic analysis

Phylogenetic trees were reconstructed using both Maximum Likelihood (ML) and Bayesian Inference (BI) approaches with both the plastid and SNP datasets, as implemented in PhyloSuite (Zhang et al., 2020). For ML analysis, IQ-TREE 2 (Minh et al., 2020) was employed. Node support was assessed through 10,000 ultrafast bootstrap (UFboot) replications. ModelFinder (Kalyaanamoorthy et al., 2017), integrated within IQ-TREE 2, was utilized for optimal base substitution model selection based on the Bayesian Information Criterion (BIC) (Luo et al., 2010). Bayesian phylogenetic trees were generated using Mrbayes 3.2.6 (Ronquist et al., 2012). The Markov Chain Monte Carlo (MCMC) parameters were set as follows: total generations (mcmcp ngen) at 3,000,000 for plastid and 10,000,000 for SNPs datasets, sampling frequency (samplefreq) at every 1,000 generation, and four simultaneous runs (nruns). The initial 25% of samples were discarded as burn-in, and the remaining samples were used to calculate posterior probabilities (PP). The ModelFinder was again used to determine the optimal base substitution model, constrained to the 24 models compatible with MrBayes (MrBayes model parameters: -m TEST -mset mrbayes). *F. pumila* was designated as the outgroup.

Phylogenetic trees derived from the chloroplast whole-genome dataset and the RADseq dataset (c90min31 dataset, as shown in **Table S3**) were compared using a mirror-image approach. Visualization was conducted in R utilizing the phytools, ape, and maps packages. Node support was categorized into high (ML/UFboots ≥ 98 or BI/PP ≥ 0.98), moderate (98 > ML/UFboots ≥ 95 or 0.98 > BI/PP ≥ 0.90), and low/no support (ML/UFboots < 95 or BI/PP < 0.90).

Another phylogenetic tree was constructed using the Tetrad tool (https://ipyrad.readthedocs.io/en/latest/API-analysis/cookbook-tetrad.html) within ipyrad, which based on the SVDQuartets algorithm of Chifman and Kubatko (Chifman and Kubatko, 2014), following a coalescent-based approach. The analysis involved all quartets, with 100 bootstrap replications. *F. pumila* served as the outgroup. This method allowed the inference of both the optimal tree and the majority-rule consensus tree, along with the calculation of branch support.

### 2.5 Analysis of population genetic structure

In this study, the population genetic structure of the *F. erecta* complex was analyzed using ADMIXTURE v1.3.0 (Alexander et al., 2009). The analysis was tailored to precisely infer the genetic structure of the *F. erecta* complex, thus, the number of inferred ancestral populations (K) was methodically varied from 2 to 18, with 1000 bootstraps using default optimization method. The determination of the optimal K value was based on the minimization of the cross-validation error (CV error). The ADMIXTURE results, including the best and second-best K values, were visualized using R.

### 2.6 Ploidy level determination

Leaf samples, dried using silica gel and weighing 20–50 mg, were processed for ploidy analysis. Each sample was finely chopped in 1 mL of LB01 buffer. Subsequently, the supernatant, containing cellular material, was gently aspirated and filtered through a 400-mesh nylon net into a 2 mL centrifuge tube. Post-centrifugation, the supernatant was discarded, and the cell pellet was stained with 80 μg/mL propidium iodide (PI) and 80 μg/mL RNase for 30 min in an ice bath. Diploid *F. pumila* (CNA0019284), with a genome size of 315.7 Mbp, sample ID 20200034_1, was utilized as an external reference (Wang et al., 2021). The stained cells samples were analyzed using a MoFlo-XDP high-speed flow cytometer (Beckman Coulter Inc., USA), operating at a sheath pressure of 60 psi with a 70 μm ceramic nozzle. Nucleic acid PI fluorescence was detected employing a solid-state laser (488 nm) and a 530-/40-nm HQ bandpass filter. Each sample was analyzed for a minimum of 100 cells to ensure statistical relevance. Samples exhibiting atypical fluorescence values underwent a minimum of two analyses to ascertain result reliability.

## 3 Results

### 3.1 Phylogenetic tree reconstruction of the complex

**Figure 2:**
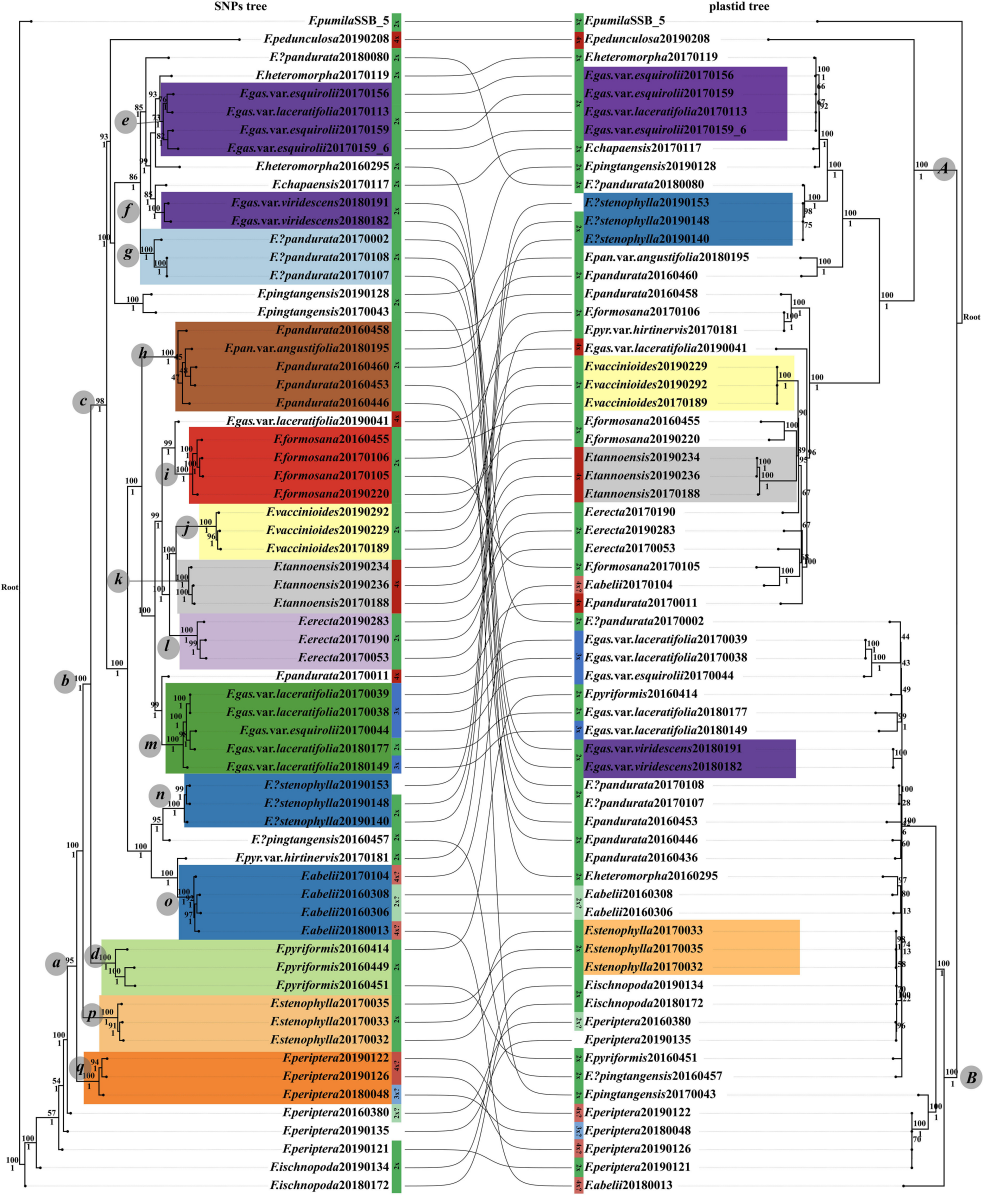
Phylogenetic relationships within the *F. erecta* complex. The left panel portrays the tree derived from the RAD c90min31 dataset, while the right panel is based on the complete chloroplast genome dataset. Branch supports are denoted as ML tree/UFboots above and BI/PP below. Sample coloration is consistent with **Figure 4**, with diploids in green, triploids in blue, tetraploids in red, and uncertain ploidy levels in lighter hues.

**Figure 3:**
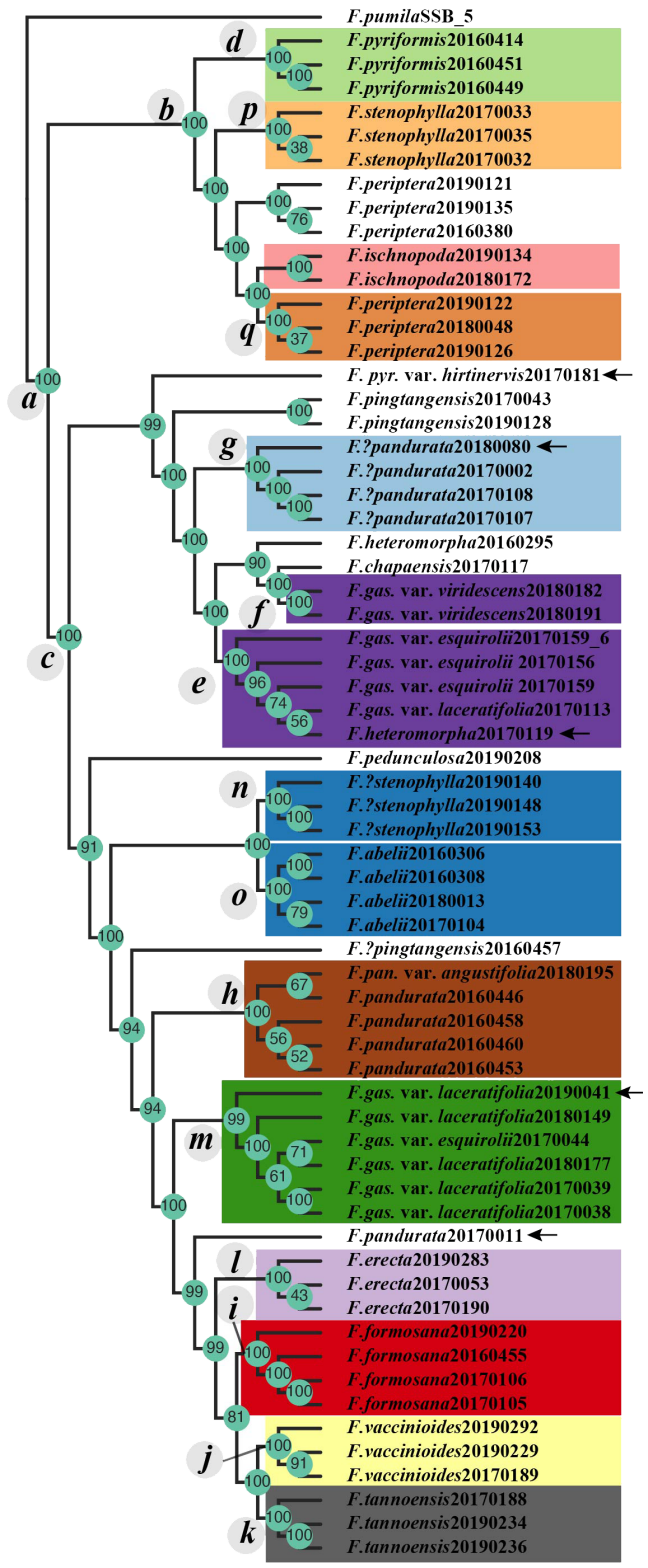
The tetrad tree of the *F. erecta* complex. Clades *c*–*q* are in alignment with those delineated in **Figure 2**. Arrows indicate the samples whose phylogenetic positioning diverges from those in the ML/BI phylogenetic tree, as depicted in **Figure 2**.

For the phylogenetic analysis, RADseq and genome skimming data were obtained from 65 samples. The RADseq dataset, specifically the c90min31 (cluster_thread 0.90 and min_sample_loci 31) unlinked-SNPs matrix, spanned a length of 33,808 bp, encompassing 8,235 parsimony-informative sites. The optimal nucleotide substitution models of this dataset for ML (Maximum Likelihood) and BI (Bayesian Inference) phylogenetic analyses, determined by the BIC (Bayesian information cirtation), were TVM+R2+F+ASC and GTR+F+ASC, respectively. The chloroplast genome dataset presented a matrix length of 137,043 bp, featuring 5,708 variable sites and 980 parsimony-informative sites. The models deemed most suitable for ML/BI analyses of this dataset, following the BIC guidelines, were K3Pu+F+I+I+R3 and GTR+F +G4, respectively. The resulting phylogenetic trees, derived from RADseq and cpDNA datasets, are illustrated in **Figure 2**. Additionally, the tetrad tree based on the RADseq data is depicted in **Figure 3**.

Most “species” in the nature are on the way to the final speciation stage. The integrative species concept combining morphology and phylogeny is used in this article. Species have unique identifiable features. The RADseq-derived phylogenetic tree exhibited robust topology, with high support values across most nodes. Clade *a* encompassed samples from almost all taxa, barring *F. ischnopoda* and partial samples of *F. periptera*. Monophyly was observed in subsets of species, such as *F. abelii* (clade *o*), *F. erecta* (clade *l*), *F. formosana* (clade *i*), *F. tannoensis* (clade *k*), and *F. vaccinioides* (clade *j*), each with uniform ML/BI support of 100/1. F*. pyriformis* (clade *d*) forms a singular branch with a support value of 100/1. However, its variety, *F. pyriformis* var. *hirtinervis*, clustered with *F. abelii* (clade *o*) under high support, sharing traits like yellowish leaf undersides and densely haired young branches and petioles. *F. pandurata* (including var. *angustifolia*) diverged into two parts: clade *h* (support value100/1), featuring typical specimens with thinner leaves and fruits lacking white surface lumps, and clade *g* (support rate 100/1), characterized by thicker leaves and fruits with white lumps. *F. stenophylla* bifurcated into two highly supported branches (clades *p* and *n*), aligning with Lu et al., 2017. Clade *p* exhibited typical leaf morphology, sparse hair, and pronounced lateral veins on the leaf underside, contrasting with clade *n* (Tengchong, Yunnan) whose morphological traits were more akin to *F. abelii* (clade *o*). The samples of the three varieties of *F. gasparriniana* (var. *esquirolii*, var. *laceratifolia* and var. *viridescens*) were segregated into three distinct clades (*e*, *f*, *m*), with support values of 73/1, 100/1, and 100/1, in which only *F. gasparriniana* var. *viridescens* is monophyly (clade *f*). Clades *e* and *f*, exhibiting lumped and densely white-pubescent fruit surfaces, respectively, were closely related to *F. heteromorpha* but distant to clade *m* (robust plants) formed a sister relationship with one sample (20170011) of *F. pandurata*. Notably, one sample (20190041) of *F. gasparriniana* var. *laceratifolia* was sister to *F. formosana* (clade *i*).

In the phylogenetic tree derived from the complete chloroplast genomes, species within the *F. erecta* complex aggregated into two principal clades, clade *A* and clade *B*, both receiving robust support values of 100/1. Notably, only *F. vaccinioides* and *F. tannoensis* independently clustered within clade *A*, each achieving a unanimous support value of 100/1. *F. stenophylla* exhibited bifurcation, embedded within clade *A* (exhibiting leaf morphology akin to *F. abelii*, with a support value of 100/1) and clade *B* (representing the archetypal *F. stenophylla*, with a support value of 98/1). In contrast to the RAD-derived phylogenetic tree, the cpDNA tree positioned the samples corresponding to clade *e* (RAD tree) within clade *A*, where they formed a sister relationship with one sample (20170119) of *F. heteromorpha* (100/1). Meanwhile, the branches corresponding to clade *f* (RAD tree, 100/1) and clade *m* (RAD tree, 100/1) were situated within clade *B*. Furthermore, the samples of *F. formosana* and *F. erecta* were dispersed throughout clade *A* without clustering, whereas the samples of *F.*?*pandurata* (corresponding to clade *g* in RAD tree), *F. periptera*, and *F. ischnopoda* resided in clade *B* yet did not form distinct clusters. Additionally, several species, such as *F. abelii*, *F. pandurata*, *F. pyriformis*, and *F. gasparriniana*, had their samples distributed across both clade *A* and clade *B*.

The tetrad tree, generated from SVDQuartets algorithm (Chifman and Kubatko, 2014) via Tetrad tool in ipyrad (Eaton and Overcast, 2020), demonstrated remarkably high bootstrap support values across its structure (**Figure 3**), dividing into three primary limbs. When compared with the gene tree derived from RADseq data (**Figure 2**, left side), the tetrad tree revealed improved clustering for certain taxa. Notably, in the gene tree, *F. pyriformis* (clade *d*) and clade *c* were sister branches, together forming a sister group to *F. stenophylla* (clade *p*), which was, in turn, sister to *F. periptera*+*F. ischnopoda*, though the latter two did not form a distinct cluster. Conversely, in the tetrad tree, *F. periptera* and *F. ischnopoda* first clustered together and then formed a sister group with *F. stenophylla* (clade *p*). This collective cluster subsequently aligned as a sister group to *F. pyriformis* (clade *d*), ultimately constituting the sister group to clade *c*. Further distinctions in the tetrad tree included the sample (20170181) of *F. pyriformis* var. *hirtinervis* positioned distantly from *F. abelii* and the sample (20170119) of *F. heteromorpha* nested within clade *e* (**Figure 3**). Additionally, the sample (20170011) of *F. pandurata* did not cluster with clade *m*. Intriguingly, the sample (20190041) of *F. gasparriniana* var. *laceratifolia* clustered within clade *m*, comprising samples from the same species, rather than with clade *i* (**Figure 3**).

### 3.2 Genetic structure analysis of the *F. erecta* complex

**Figure 4:**
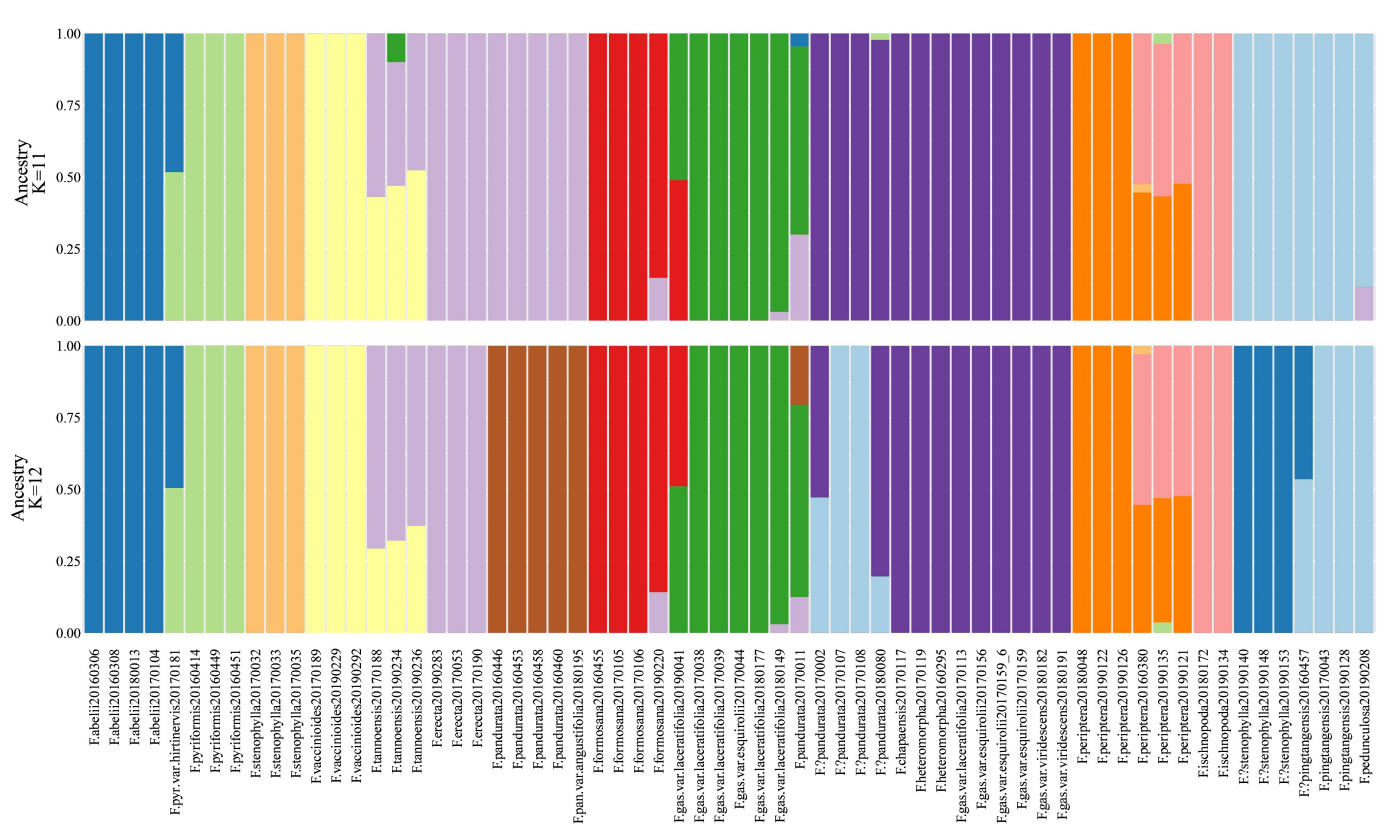
ADMIXTURE results for K values of 11 and 12.

CV error metrics (**Table S4**, **Figure S1**) identified 11 as the best-fit number of genetic clusters (K), with 12 as a secondary optimum (**Figure 4**). At K=11, distinct genetic backgrounds were evident for several species, including *F. abelii*, *F. pyriformis*, *F. stenophylla*, *F. vaccinioides*, and *F. formosana*, indicating well-defined genetic separations (**Figure 4**). Notably, *F. erecta* and *F. pandurata* samples displayed a shared genetic background (light purple), yet at K=12. The samples of *F. gasparriniana* (as depicted in clades *e*, *f* in **Figures 2**, **3**), *F.*?*pandurata*, *F. heteromorpha*, and *F. chapaensis* shared a uniform genetic background (dark purple), hinting at close genetic relationships. Intriguingly, *F.*?*stenophylla*, morphologically akin to *F. abelii*, exhibited a genetic background significantly divergent from *F. stenophylla*. The genetic make-up of *F. tannoensis* samples was notably heterogeneous, reflecting an admixture of genetic contributions from *F. vaccinioides* and *F. erecta*. Furthermore, the genetic profiles of three *F. periptera* samples from Nonghua, Guangxi (20160380, 20190135), and Malipo, Yunnan (20190121) suggested hybrid origins. Additional samples exhibiting mixed genetic backgrounds included *F. pyr*. var. *hirtinervis*20170181, *F. gas*. var. *laceratifolia*20190041, *F. pandurata*20170011, and *F. formosana*20190220 (**Figure 4**).

### 3.3 Analysis of chromosomal ploidy levels in the *F. erecta* complex

**Figure 5:**
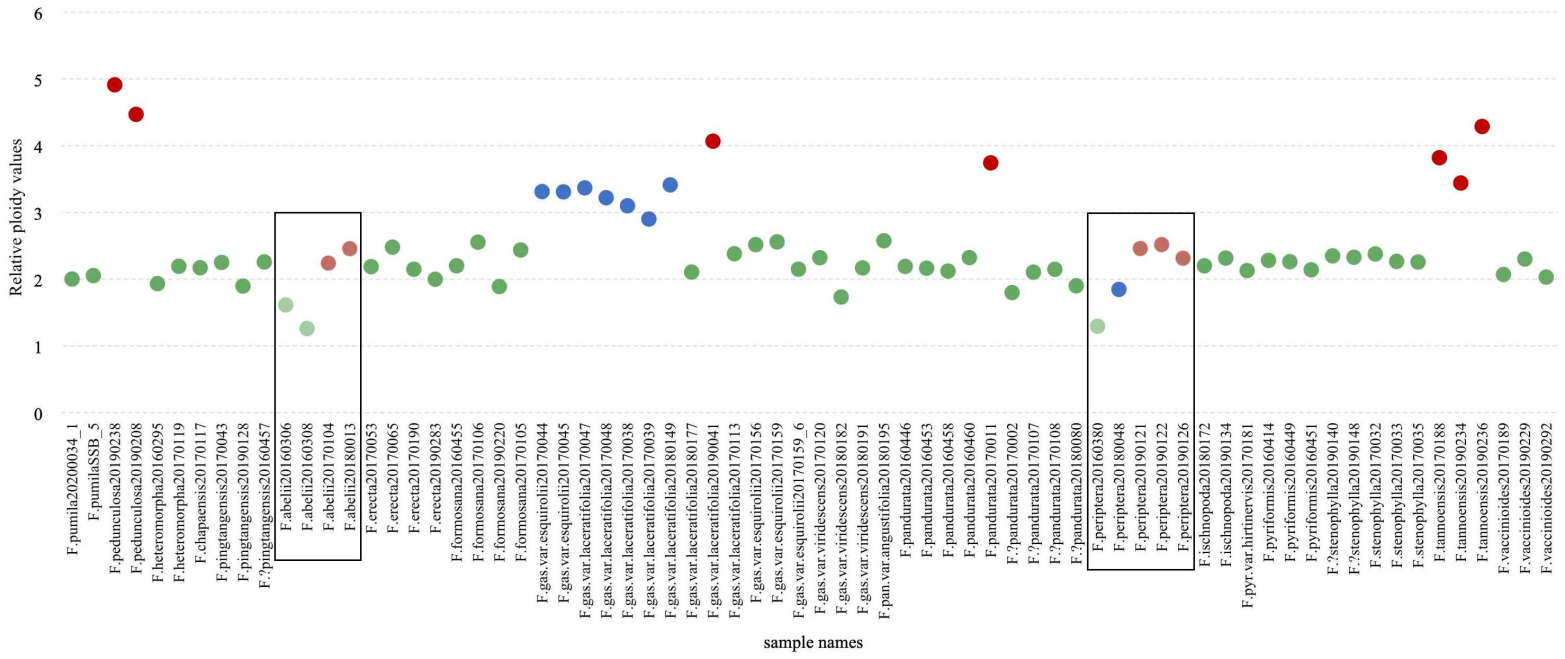
Relative ploidy values of the *F. erecta* complex. The y-axis represents the relative ploidy values, and the x-axis lists the sample names. The color coding (green for diploids, blue for triploids, and red for tetraploids) facilitates easy identification of the ploidy level, with lighter shades indicating uncertain ploidy.

A comprehensive ploidy level analysis was conducted on 68 samples of the *F. erecta* complex, yielding 98 valid measurements (**Table S5**). To ensure data consistency, some samples were subjected to multiple tests. The number of cells counted per sample ranging from 95 to 4,746 in individual assays. Relative ploidy levels were calculated by comparing the average fluorescence mean values of each sample with those of the diploid reference species, *F. pumila* (CNA0019284). **Figure 5** graphically represents these relative ploidy values. The majority of the *F. erecta* complex samples were diploid, such as *F. pandurata* (clade *h*, **Figure 2**), *F.*?*pandurata* (clade *g*), *F. stenophylla* (clade *p*), and *F.*?*stenophylla* (clade *n*). *F. gasparriniana* exhibited a complex ploidy pattern, with 2×, 3×, and 4× levels observed (**Figures 2**, **5**). Samples from clade *e* and clade *f* (**Figure 2**) were consistently diploid, while clade *m* samples exhibited 2× and 3×, predominantly triploid. An unclustered sample (20190041) was identified as tetraploid. The chromosome ploidy of *F. abelii* and *F. periptera* was quite unique, with their relative ploidy values significantly lower than other species. The first one, *F. abelii* displayed two ploidy levels, with relative values of 1.26 and 2.46, suggestive of a 1:2 ratio. The second, *F. periptera* exhibited three distinct ploidy levels with relative values of 1.29, 1.84, and 2.51, approximating a 2:3:4 ratio. Whereas *Ficus tannoensis* and *F. pedunculosa* exhibited an approximate 4× ploidy level (**Figure 5**).

## 4 Discussion and conclusion

### 4.1 Morphological variation and genetic differentiation in the *F. erecta* complex

**Figure 6:**
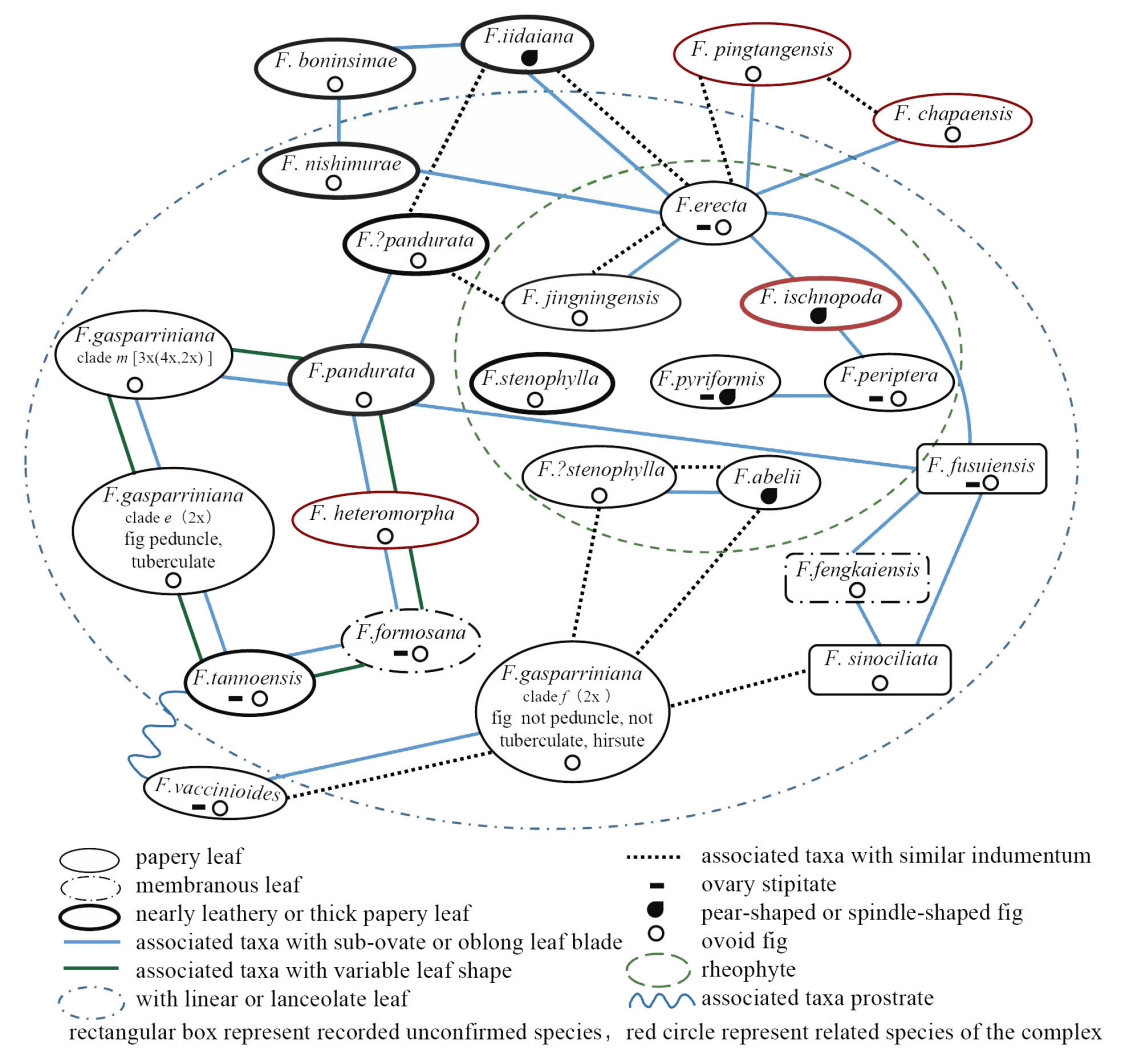
Morphological relationships among various taxonomic units of the *F. erecta* complex and its close relatives.

The *F. erecta* complex is characterized by substantial intraspecific variability and indistinct interspecific boundary, leading to pervasive morphological overlap (**Figure 6**, which were integrated from type specimens, botanical descriptions, actual observations, and comparative taxonomic anatomy). The syconia (fig fruits) of the complex are generally uniform in morphology and size (1–2 cm), with inflorescences varying in shape from spherical to pear-shaped or spindle-shaped. Typically, the peduncles and ovary stalks exist or not. The lack of significant morphological variation among inflorescences and uniform phenological characteristics render leaf shape variations pivotal in species circumscription within the complex. However, reliance on foliar features alone often proves insufficient for accurate species identification.

Several widespread taxa within the complex, exemplified by *F. erecta、F. formosana* and *F. pandurata*, display considerable leaf shape polymorphism, mirroring similar variation patterns. Leaves predominantly range from elliptical to inversely elliptical, with an aspect ratio of less than 3, but gradually transition to elongated forms with aspect ratios exceeding 10 and vertical side veins (**Figure 6**, green and blue lines). Some leaves also exhibit split tips. Comparable variations are observed in closely related species like *F. heteromorpha*, *F. pingtangensis*, *F. chapaensis*, and *F. daimingshanensis*. Typical rheophytic species in the complex, including *F. abelii*、*F. pyriformis*、*F. peritera*、*F. ischnopoda* and *F. stenophylla*, share lanceolate leaf and sparse indumentum (exception: *F. abelii*), with elongated peduncle. However, these characteristics exhibited inconspicuous interspecific variation among them (**Figure 6**, green dotted line). *F. gasparriniana*, comprising multiple varieties, display the most pronounced variation, with *F. gasparriniana* var. *esquirolii* and var. *laceratifolia* primarily differentiated by leaf apex division or not. *F. gasparriniana* var. *viridescens*, with prominent indumentum, contrasts significantly from other varieties. Simultanously, these varieties of *F. gasparriniana* also shares morphological features with other groups in the complex. Additionally, differentiation in leaf texture and indumentum is evident within the complex, varying from papery to leathery or membranous and from dense to glabrous, yet these are insufficient for distinguishing the numerous taxonomic units. Therefore, careful consideration is necessary when identifying groups based on leaf morphology, demanding a comprehensive understanding of the group’s variation range (Keay, 1998).

**Figure 7:**
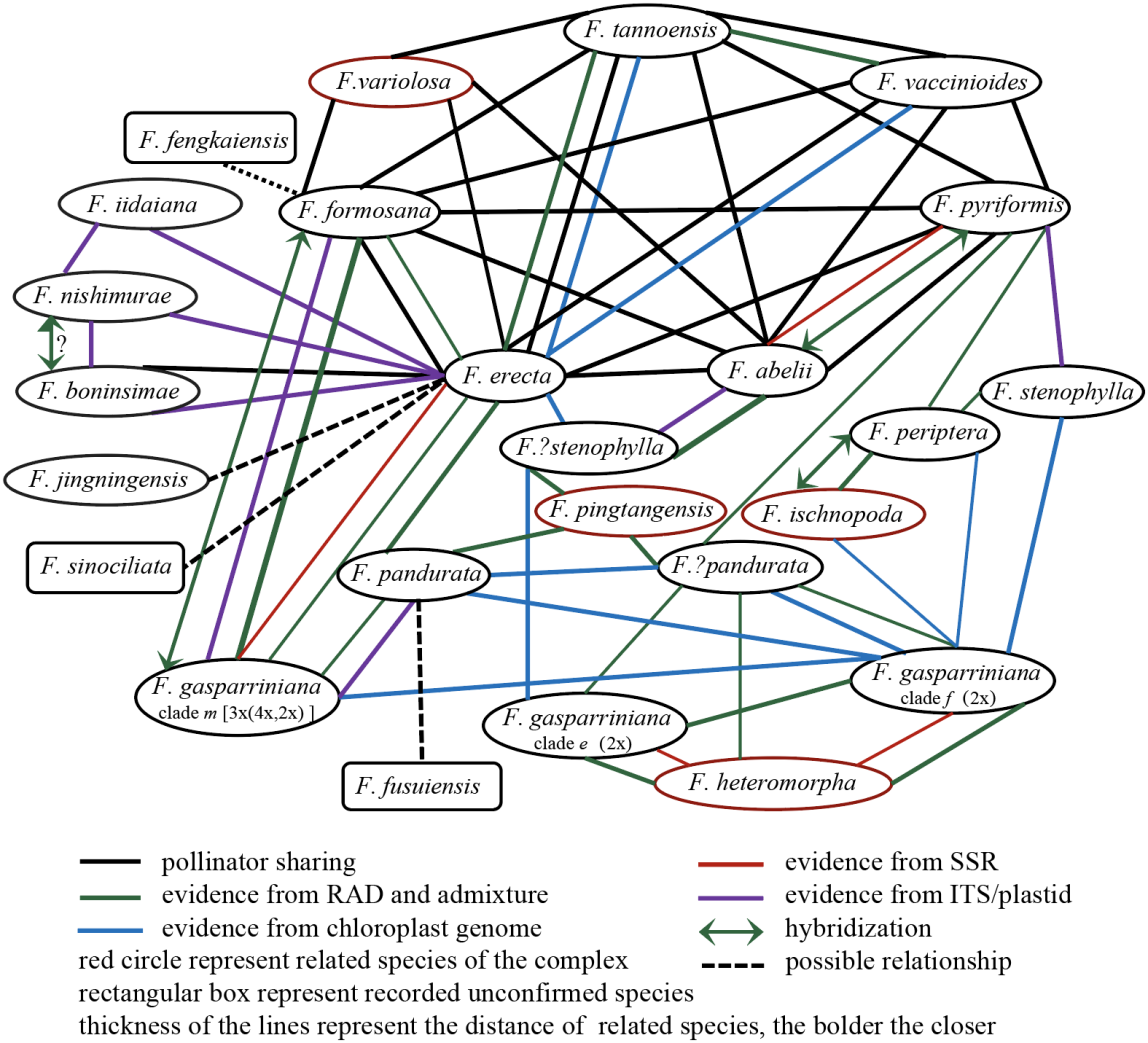
Genetic relationships among various taxonomic units within the *F. erecta* complex.

Species within the *F. erecta* complex often exhibit sympatric or parapatric distributions, conducive to frequent gene flow (Whittemore and Schaal, 1991; Wang et al., 2021). Most species in the complex have similar ostiole sizes and overlapping fruiting phenology, facilitating the sharing of pollinating fig wasps. In extensively studied regions like Southeast China, pollinator sharing is commonplace. For instance, in Hong Kong, the pollinators of *F. pyriformis* and *F. variolosa* are almost indistinguishable (Hill, 1967), in Taiwan, *F. formosana* and *F. vaccinioides* share the pollinator *Blastophaga nipponica* (Wachi et al., 2016), and in Guangdong, five species including *F. erecta*, *F. formosana*, *F. abelii*, *F. pyriformis* and *F. variolosa* share the pollinator *Blastophaga silvestriana* (Su et al., 2022), leading to frequent hybridization within the *F. erecta* complex. Hybridization is also observed between closely related species like *F. boninsimae* and *F. nishimurae* (Yokoyama, 2003) (**Figure 7**, a summary of genetic relationships based on existing molecular evidence, including published results and the results of this study).

According to our study (**Figures 2**–**4**), high-frequency hybridization or gene flow events were also evident in taxa from other geographical areas, such as between *F. abelii* and *F. pyriformis*, *F. periptera* and *F. ischnopoda*, and *F. formosana* and *F. gasparriniana* in Southwest China. Both phylogenetictrees from SNPs datasets displayed several individuals in unusual positions (e.g., *F. pyr*. var. *hirtinervis*20170181, *F. pandurata*20170011), whose phylogenetic misplacement typically aligned with hybridization events (**Figures 2**, **3** (Bruun-Lund et al., 2017)) or genome polyploidization (**Figure 5**). Moreover, multiple ploidy levels were observed within several species (e.g., *F. abelii*, *F. gasparriniana*, *F. pandurata* and *F. periptera*). These findings collectively indicate blurred interspecific genetic boundaries in the *F. erecta* complex, leading to the formation of a tightly-knit species network (**Figure 7**). Nonetheless, there was a notable lack of alignment between the morphological relationship network (**Figure 6**) and the molecular systematic network (**Figure 7**). This divergence may be attributed to the limits of phylogenetic trees, which are impacted by gene incongruence arising from hybridization, horizontal gene transfer, and gene duplication and loss (Maddison, 1997; Massatti et al., 2016). The influence of phenotypic plasticity (West-Eberhard, 1989; Duminil and Di Michele, 2009; Fujita et al., 2012; Prata et al., 2018) and convergent evolution (Losos, 2011) may also contribute to these complex patterns.

### 4.2 Expansion of the *F. erecta* complex to encompass the entire subsect. *Frutescentiae*

The *F. erecta* complex was originally delineated based on a phylogenetically well-supported branch encompassing 11 taxonomic groups, augmented by selected cryptic species and island endemics, *as per* morphological characteristics (Lu et al., 2017). Lu et al., however, failed to establish a stable phylogenetic structure for taxa external to the complex, hinting at its potential species inclusions (Lu et al., 2017). Notably, *F. gasparriniana* is deemed to a member of the complex, while its variety, *F. gasparriniana* var. *esquirolii*, originally classified outside the complex in the study of Lu et al. In addressing this, our study incorporated additional samples from *F. gasparriniana* var. *esquirolii*, *F. ischnopoda*, *F. heteromorpha*, *F. pingtangensis*, *F. chapaensis* and *F. pedunculosa*, revealing their integration within the complex (**Figures 2**, **3**). Specifically, *F. gasparriniana* var. *esquirolii* and *F. heteromorpha* clusters closely with other *F. gasparriniana* varieties (clade *f*), and *F. pandurata* (**Figure 2**, clade *e*, **Figure 3**, clade *e*). The substantial gene flow among these species confirmed their inclusion in the *F. erecta* complex. The affiliation of *F. periptera*, an established member of *Ficus erecta* complex, with the geographically and ecologically aligned *F. ischnopoda*, coupled with observed hybridization at the type locality (samples *F. periptera*20160380, *F. periptera*20190121 and *F. periptera*20190135), further supports expanding the complex’s scope (**Figure 4**). Moreover, *F. ischnopoda*’s alignment with the complex’s phylogenetic tree suggests its inclusion.

While the study did not encompass all related taxa in the subsect. *Frutescentiae*, such as *F. oleifolia*, *F. variolosa*, *F. filicauda*, and *F. trivia*, the phylogenetic proximity of the newly incorporated samples (e.g., sister group relationships) to these species had been evidenced in the previous study (Lu et al., 2017), notably the integration of *F. pedunculosa* (**Figures 2**, **3**), previously deemed a sister group to the remaining subsection (Lu et al., 2017). Thus, it is worth believing that there is no clear genetic gap between the complex and allies out of the complex. Besides, there is no obvious morphological difference between the complex and allies out of the complex according to comparative taxonomic anatomy (Gao, 2018). Consequently, the *F. erecta* complex should be broadened to include the entire subsect. *Frutescentiae*. The previous studies have identified subsect. *Frutescentiae* as a robust monophyletic group within the genus *Ficus*, characterized by their dioecious syconia, predominantly small shrub-like habit, the distinct Terminalia-branching pattern, axillary figs, staminate flowers mostly scattered or near the ostiole,stamens 2 or 3, representing one of its nine primary branches (Berg, 2003; Gardner et al., 2023). Addressing taxonomic issues within any individual species of the complex necessitates a comprehensive understanding of the genetic backdrop of the entire subsection, highlighting the intertwined evolutionary narratives within this group.

### 4.3 Widespread polyploidy in the *F. erecta* complex

Our study uncovered the prevalence of polyploidization within the *F. erecta* complex. For instance, *F. gasparriniana*, along with its varieties, demonstrated a range of ploidy levels (2×, 3×, 4×). Tetraploidy has been detected in *F. pandurata* (*F. pandurata* 20170011), *F. tannoensis* and *F. erecta*. Relatively low nucleic acid PI fluorescence value together with a 1:2 ratio suggests possible diploidy and tetraploidy but with a smaller genome size in *F. abelii* (**Figure 5**). Similar low absolute value in *F. periptera* presumed to exhibit 2×, 3×, and 4× ploidy levels. Due to limited sampling in ploidy determination, other species within the complex may exhibit undetected polyploidy.

The peculiar phylogenetic positions of certain individuals, such as *F. pandurata*20170011 and *F. gasparriniana* var. *laceratifolia*20190041, which did not cluster with other samples of their species (**Figures 2**, **3**), can be attributed to their allopolyploid nature. This phenomenon exemplifies the role of allopolyploidization in promoting rapid species differentiation (Yang et al., 2023) and complex evolution (Hörandl, 2022; Karbstein et al., 2022; Yang et al., 2023). The genome duplication of unreduced gametes from diploids plants through a triploid bridge is a key pathway of polyploids, as demonstrated by triploids in *F. gasparriniana* (clade *m*) and *F. periptera* (**Figures 2**, **5**), suggesting ongoing polyploid formation in these taxa. Polyploidy occurring within multiple species resulting in species with multiple ploidy levels. Furthermore, considering polyploidization and/or hybridization often coexist with apomixis (Hojsgaard and Hörandl, 2019; Hörandl, 2022), the presence of apomixis in species like *F. abelii*, *F. erecta*, *F. gasparriniana* (clade *m*), *F. pandurata*, *F. pedunculosa*, *F. periptera*, and *F. tannoensis* is in need of further investigation. Our unpublished observations indicate the apparent occurrence of apomixis in *F. gasparriniana* (clade *m*).

### 4.4 Phylogenetic relationships among taxa and taxonomic resolution in the *F. erecta* complex

#### 4.4.1 *Ficus gasparriniana* contains at least three independent lineages

Phylogenetic analyses revealed the subdivision of *F. gasparriniana* into three distinct clades, designated as clades *e*, *f*, and *m*, which do not correspond directly with the three recognized varieties of the species. Clades *e* and *f* exhibit closer phylogenetic affinity, contrasting with the relatively distant clade *m*. The ploidy levels within these clades vary, with clades *e* and *f* being diploid and clade *m* predominantly triploid with containing diploid individuals. Morphologically, clade *e* parallels *F. heteromorpha*, characterized by thin papery leaves, fruits with white lumps on the surface, and reddish-brown young branches and petioles. In contrast, clade *f* exhibits leafs densely covered with soft white hair, fruits similarly enveloped with soft white hair devoid of lumps, and green young branches and petioles, underscoring clear morphological distinctions between these two clades. The cpDNA-based phylogenetic tree segregates clade *e* within clade *A*, whereas clades *f* and *m* are nested within clade *B*, suggesting substantial genetic divergence between clades *e* and *f.* Clade *m* not only differs genetically and in ploidy levels but also manifests larger plant, leaf, and fruit sizes, coupled with evidence of apomixis, markedly distinguishing it from the former clades. Given the absent samples of *F. gasparriniana* var. *gasparriniana*, it can be inferred that *F. gasparriniana* encompasses at least three independent lineages, confirming its status as a polymorphic species. However, the redefinition of *F. gasparriniana* is contingent upon an extensive elucidation of the phylogenetic intricacies within the complex. This revelation about *F. gasparriniana* underscores the complex taxonomic intricacies within the *F. erecta* complex. The presence of distinct genetic lineages and morphological variabilities necessitates meticulous and comprehensive investigations for accurate species delineation and an in-depth understanding of their evolutionary histories.

#### 4.4.2 *Ficus pandurata* contains two lineages

*Ficus pandurata* bifurcated into two distinct lineages: clade *h* and clade *g*, as revealed through RADseq-based analysis, both exhibiting diploid chromosomal structures (**Figure 2**). Genetic structuring analysis indicated that clade *h* shared a genetic affinity with *F. erecta*, while clade *g* aligned more closely with the genetic backgrounds of *F. gasparriniana* (clades *e* and *f*) and *F. heteromorpha*. A detailed morphological assessment revealed divergent characteristics between these clades. Clade *h*, encompassing *F. pandurata* and its varieties, is characterized by a thinner leaf texture and an absence of white lumps on the fruit surface. Conversely, clade *g* exhibits a thicker leaf morphology and typically bears fruits with tuberculate surfaces, delineating clear morphological distinctions from *F. pandurata* and its varieties, thus clade *g* represents a hitherto undescribed cryptic species within the *F. erecta* complex.

#### 4.4.3 “*Ficus stenophylla*” samples from Tengchong, Yunnan (clade *n*), potentially represent a novel species

In this study, samples identified as *F. stenophylla* from Tengchong, Yunnan (clade *n*), exhibit a distinct phylogenetic lineage in the RADseq-derived tree, divergent from the canonical *F. stenophylla* (clade *p*) and showing closer affinity to *F. abelii*. This observation corroborates the findings of Lu et al (Lu et al., 2017), which utilized two nuclear markers (ITS+ETS). In the cpDNA phylogeny, *F. abelii* samples are distributed across Clade *A* and Clade *B*, whereas the *F. stenophylla* samples from clade *n* and clade *p* cluster distinctly in Clade *A* and Clade *B*, respectively (**Figure 2**). ADMIXTURE analysis at K=11 reveals divergent genetic compositions between two clades of *F. stenophylla* (*n* and *p*) and *F. abelii* (**Figure 4**). Morphological examination of clade *n* samples reveals an inverted ovate-lanceolate leaf shape, subdued lateral veins on the lower surface of blade, and elongated elliptical and often dehiscent syconia, bearing resemblance to *F. abelii*. Conversely, the prototypical *F. stenophylla* (clade *p*) displays linear-lanceolate leaves with pronounced lateral veins on the lower surface and slightly pubescent, elliptical to spherical syconia. These morphological distinctions, coupled with the phylogenetic and genetic data, strongly suggest that clade *n* represents a hitherto undescribed cryptic species within the *F. erecta* complex.

#### 4.4.4 Discussion on the species status in other taxa of the *F*. *erecta* complex

The four species, *F. tannoensis*, *F. vaccinioides*, *F. erecta*, and *F. formosana*, each formed distinct monophyletic groups on both the RADseq-based phylogenetic trees (**Figures 2**, **3**), constituting a bigger branch with strong support (100/1). This clustering suggests that they are closely related yet independent species. Notably, *F. tannoensis* exhibits hybrid origins, sharing genetic backgrounds with *F. vaccinioide* and *F. erecta* (**Figure 4**, K=11, K=12). Morphologically, *F. tannoensis* shares rare liana traits with *F. vaccinioides*, both native to southern Taiwan in China. However, its leaf shape variation is strikingly similar to *F. erecta*, albeit with noticeably smaller leaves. This is potentially influenced by its other parent, *F. vaccinioides*, which possesses the smallest leaves in the complex. Phylogenetically, *F. tannoensis* is positioned between *F. vaccinioide* and *F. erecta*. Furthermore, ploidy analysis confirmed tetraploidy in entire *F. tannoensis* samples in this study. Thus, it is plausible that *F. tannoensis* represents an allopolyploid species derived from *F. vaccinioide* and *F. erecta*, a finding that contributes novel insights into speciation processes within *Ficus* and highlights allopolyploidization as a mechanism driving its rich species diversity.

In the RADseq-derived phylogenetic tree, samples of *F. ischnopoda* and *F. periptera* failed to coalesce into monophyletic clusters. Intriguingly, some specimens from these taxa aligned as sister groups to other divergent clades within the complex (**Figure 2**). In stark contrast, the tetrad tree, constructed utilizing the SVDQuartets algorithm, presented a unified, well-supported branch comprising samples of both *F. ischnopoda* and *F. periptera* (**Figure 3**). Considered in light of the SVDQuartets algorithm is proficiency in resolving phylogenetic structures amid gene flow scenarios (Long and Kubatko, 2018), the tetrad tree topology should more accurately mirror the true evolutionary history of these taxa. This conjecture was further corroborated by the evident genetic background sharing between *F. ischnopoda* and *F. periptera* (**Figure 4**). Ploidy analysis underscores the occurrence of polyploidy phenomena (3× and 4×, **Figure 5**) exclusive to *F. periptera*. Coupled with the taxonomic challenge posed by their low morphological distinctiveness, the taxonomic statuses of these two species necessitates more rigorous scrutiny.

Previous molecular phylogenetic analyses suggested that *F. iidaiana*, *F. nishimurae*, and *F. boninsimae*, endemic to Japan’s Ogasawara Islands, exhibited close phylogenetic relationships with *F. erecta* (Azuma et al., 2010) *F. iidaiana* stands out with its larger stature, longer lamina and petioles, and more elongated fruits compared with *F. nishimurae* and *F. boninsimae* (Yokoyama and Iwatsuki, 1998; Yokoyama, 2006). It is also unique as the sole tetraploid among these species (Ono, 1990). *F. nishimurae* and *F. boninsimae*, while morphologically similar, exhibit divergences in some quantitative traits (such as leaf and fruit sizes) and habitat preferences. Notably, hybridization events between these two species have been documented (Kusumi et al., 2012). Despite their morphological similarities and instances of hybridization, each species maintains distinct characteristics, justifying their separate taxonomic statuses. However, this research did not include samples from these species, indicating a gap in comprehensive data that could further clarify their biogeographic histories within the complex.

*Ficus fusuiensis* resembles some variant individuals of *F. pandurata*, but *F. fusuiensis* can be distinguished by its cuneate leaf base and ovate-lanceolate leaf shape. Similarly, *F. fengkaiensis* and *F. formosana* demonstrate morphological parallels, yet *F. fengkaiensis* is characterized by broader leaves, elongated peduncle within the flowers. *F. sinociliata* closely resembles a variant form of *F. erecta* var. *erecta*. The primary distinguishing features of *F. sinociliata* include prominently marginal ciliate and reduced fruit size. It is noteworthy that only type specimens of these three species have been documented to date (Chang, 1983, Chang, 1984), with a lack of subsequent collection records. The observed high morphological variability, combined with extensive hybridization and the occurrence of polyploidization within the *F. erecta* complex, raises the possibility that these species may represent either hybrid progeny or occasional morphological variants of related species within the complex.

Chen et al. (Chen et al., 2020). recently described *F. jingningensis*, a novel species within the *F. erecta* complex. It features narrow lanceolate leaves with a cordate base based on both type specimens and our collectings from the type locality. However, a close comparison of leaf texture, indumentum patterns, and fruit morphology reveals a striking resemblance to *F. erecta*, suggesting that *F. jingningensis* may represent a specialized morphological variant within this species.

### 4.5 *Ficus erecta* complex is a rare and huge evolutionally joint species group with frequent reticulate evolution and polyploidization

Traditional molecular markers, typically sourced from singular organelles or limited nuclear DNA regions (Freeland et al., 2015), often fall short in reconstructing complex reticulate relationships due to constraints like uniparental inheritance, heterogeneous evolutionary history of the markers, and/or low variability. These challenges have historically hindered the precise delimitation of species within complexes (Rittmeyer and Austin, 2012; Kirschner et al., 2015; Rothfels, 2021).

This study, expanding upon Lu et al. (2017), leveraged vast cpDNA and RADseq data to amass a robust set of genetic markers. The resultant phylogenetic tree of the *F. erecta* complex exhibited pronounced nuclear-cytoplasmic conflicts (**Figure 2**) and notable discordances between SNPs trees which based on different tree construction methods (**Figures 2**, **3**). This analysis not only identified multiple species in the complex with hybrid origins but also unraveled intricate patterns of reticulate evolution (**Figure 7**), thereby elucidating the phylogenetic relationships and taxonomic statuses of some taxa within the complex.

These phylogenomic instances evidence the faciliation of extensive RAD and cpDNA sequencing data in reconstructing phylogenetic relationships, identifying cryptic species, and resolving the complex evolutionary histories of species or taxonomic groups (Maddison, 1997; Zhou et al., 2018; Spriggs et al., 2019; Zhou et al., 2020). Additionally, this approach contributes to our understanding of the origins and evolutionary histories of polyploid species (He et al., 2023). Nevertheless, the study reveals challenges in achieving complete clustering of certain species on the phylogenetic trees, with several branches exhibiting low support values (**Figure 2**), and an overall lack of enhanced species resolution. This highlights that the greater the variability encompassed within a complex and the more comprehensive the sampling strategy, the more likely it is to reveal transitional morphological characteristics, hybridization events, and polyploidization phenomena. These factors collectively contribute to blurred species boundaries and amplified complexity in interspecific relationships (Duminil and Di Michele, 2009). Such scenarios underscore the conceptualization of a species complex as an assembly of interconnected and interdependent populations, each exhibiting unique morphological or genetic traits, yet demonstrating a continuum of variation across populations (Derkarabetian and Hedin, 2014; Hori et al., 2014). Extreme instances like the *F. erecta* complex, encompassing dozens of taxa, remain relatively rare. Future research, leveraging comprehensive sampling and population genomic data, is essential to elucidate the complex’s evolutionary history, speciation mechanisms, and species delineation. Such endeavors represent a contemporary approach to the longstanding biological challenge of species classification in the modern scientific era.

## Data availability statement

The datasets presented in this study can be found in online repositories. The raw data for *Ficus erecta* complex generated for this study can be found at NCBI https://www.ncbi.nlm.nih.gov/sra/PRJNA1059135. The names of the repository/repositories and accession number(s) can be found in the article/**Supplementary Material**.

## Author contributions

XW: Formal analysis, Methodology, Software, Writing – original draft, Writing – review & editing. SL: Investigation, Resources, Supervision, Validation, Funding acquisition, Writing – review & editing. ZZ: Resources, Software, Writing – review & editing, Investigation. JZ: Resources, Validation, Writing – review & editing. LM: Data curation, Writing – review & editing. HL: Funding acquisition, Project administration, Resources, Supervision, Writing – original draft, Writing – review & editing.
