# Supplementary figures and images for: Hybridization, polyploidization, and morphological convergence make dozens of taxa into one chaotic genetic pool: a phylogenomic case of the Ficus erecta species complex (Moraceae)
Source: Front Plant Sci. 2024 Mar 26;15:1354812. doi: 10.3389/fpls.2024.1354812 (PMC11002808; doi:10.3389/fpls.2024.1354812)

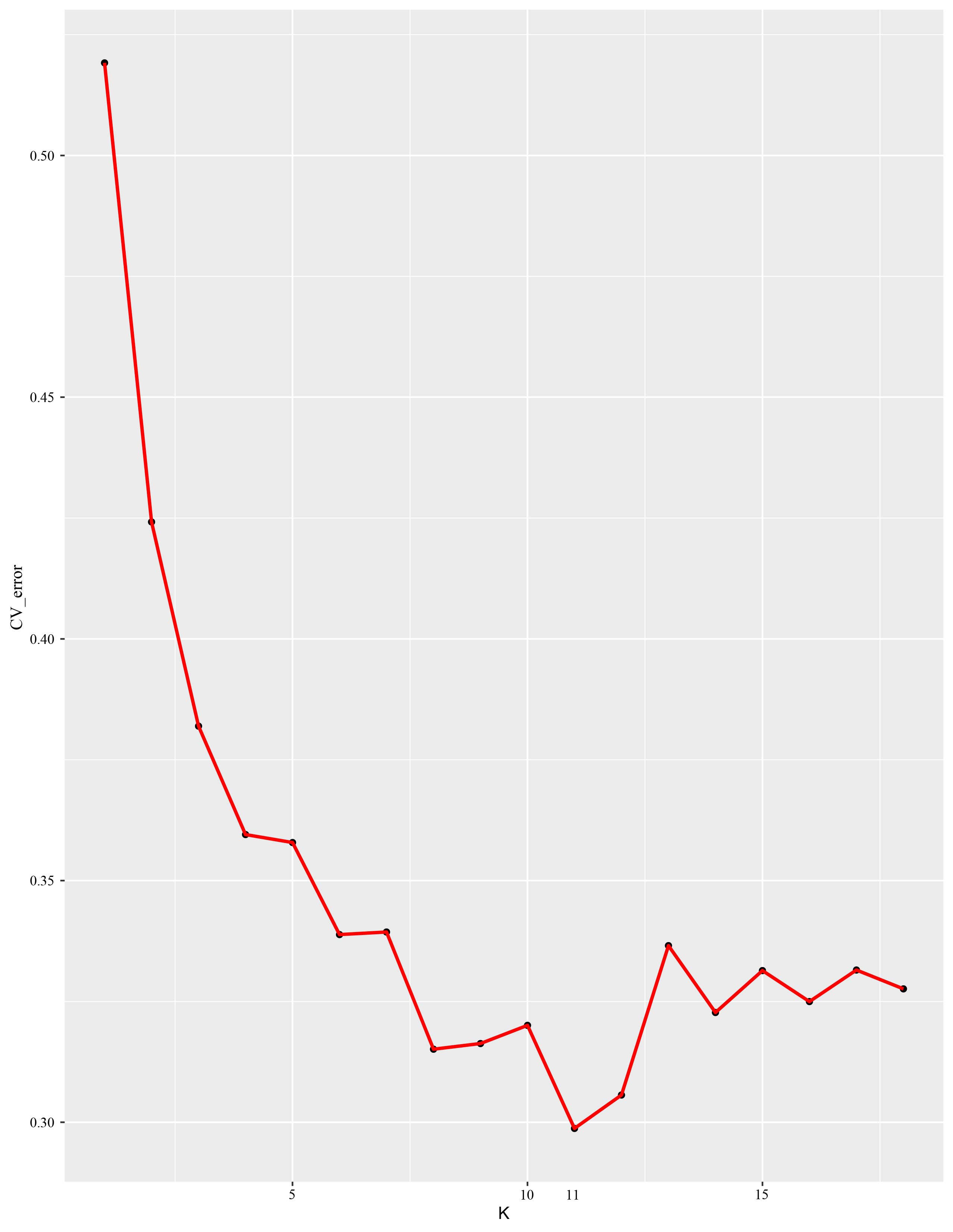

Supplement: Supplementary file 1 [file Image_1.jpeg]
